# Evaluating Stress during Pregnancy: Do We Have the Right Conceptions and the Correct Tools to Assess It?

**DOI:** 10.1155/2018/4857065

**Published:** 2018-02-01

**Authors:** Raquel González-Ochoa, Elly N. Sánchez-Rodríguez, Anahi Chavarría, Gabriel Gutiérrez-Ospina, Tania Romo-González

**Affiliations:** ^1^Biología y Salud Integral, Instituto de Investigaciones Biológicas, Universidad Veracruzana, Xalapa, VER, Mexico; ^2^Instituto de Investigaciones Psicológicas, Universidad Veracruzana, Xalapa, VER, Mexico; ^3^Departamento de Medicina Experimental, Facultad de Medicina, Universidad Nacional Autónoma de México, Ciudad de México, Mexico; ^4^Departamento de Biología Celular y Fisiología, Instituto de Investigaciones Biomédicas, Coordinación de Psicobiología, Facultad de Psicología, Universidad Nacional Autónoma de México, 04510 Ciudad de México, Mexico

## Abstract

Gestational stress is believed to increase the risk of pregnancy failure and perinatal and adult morbidity and mortality in both the mother and her child or children. However, some contradictions might arise from methodological issues or even from differences in the philosophical grounds that guide the studies on gestational stress. Biased perspectives could lead us to use and/or design inadequate/incomplete panels of biochemical determinations and/or psychological instruments to diagnose it accurately during pregnancy, a psychoneuroimmune-endocrine state in which allostatic loads may be significant. Here, we review these notions and propose a model to evaluate and diagnose stress during pregnancy.

## 1. Introduction

According to conventional wisdom, gestational stress increases the risk of pregnancy miscarriages and predisposes the mother to perinatal infections, premature labor, hemorrhages and preeclampsia [1–12]. Children are also presumed to be negatively affected by prenatal stress since it predisposes them to develop mood disorders, attention deficit disorder, perinatal infections, and obesity at early ages and cancer and/or degenerative disorders in adulthood (e.g., cardiovascular disease, cancer, diabetes, obesity, and behavioral, cognitive, and mood disorders) [13–18]. All of these are major sociomedical problems that form part of current health political agendas in many countries worldwide. Nevertheless, in spite of broad acceptance of these concepts, recent research consistently fails to confirm such premises; Table 1 shows a list that clearly exemplifies the contradictions in the literature.

Although discrepancies among studies might be attributed to inappropriate statistical designs [19] or even to methodological flaws (Table 1), they might also reflect conceptual differences among the perspectives that scientists with dissimilar backgrounds have on stress. In turn, biased perspectives might lead us to use and/or design inadequate/incomplete panels of biochemical determinations and/or psychological instruments to diagnose it. This circumstance is more likely to occur when assessing stress in pregnant women because pregnancy itself imposes stressful allostatic loads on the mother that nonpregnant woman do not have. Indeed, pregnant women are known to be more emotionally vulnerable, to develop ambivalent feelings, or to have concerns about the future and/or about their ability to cope with the social demands of motherhood [20–22]. In sum, the stress found in pregnancy may be stronger than ongoing everyday stress since there is a greater association between the psychological status and the physiological responses [5].

Hence, in this review, we begin with a brief comment on the historical background that led us to define stress as a process in which the body, the mind, and the sociocultural status are disarticulated; afterwards we describe the negative consequences that this had on its accurate diagnosis. We then propose an integrated model of stress in which all these elements are considered within the same conceptual framework. The proposed model also ponders the accumulated life stress and epigenetic inheritance as conditioning factors of each individual's stress response. We then analyze the use of this model to better diagnose chronic stress during pregnancy, considering pregnancy itself as an allostatic stress process. Finally, a protocol is proposed in order to diagnose chronic gestational stress based upon the combined use of physiological and psychological tests. It is worth mentioning that our reflection is aimed at understanding the stress response of pregnant women during common life circumstances. Other extreme settings such as those associated with natural disasters, war, or terrorist attacks that might affect pregnant women are not considered [5, 23–25], although we recognize that these types of events may take the pregnant woman body's abilities to restore homeostasis to its limits. Lastly, this review must not be expected to be an erudite compilation and discussion of the literature, but rather a reflection aimed at creating a rational framework that could unify criteria for a more systematic study of gestational stress in the future.

## 2. Stress Models: A Historical Breviary and Their Consequences

As mentioned in the introductory remarks, recent research on gestational stress has led to a conundrum. Whereas most research supports the notion that gestational stress upsets the health of the mother and her child before, during, and after parturition, other reports claim the opposite. In addition, most research on the topic has found an association between preterm birth, delayed baby's development, preeclampsia, and low birth weight and stress. However, this very literature does not clearly indicate whether health problems might indeed be associated with stress and/or anxiety and depression since the psychological psychometric instruments used are not designed to estimate stress.

We believe that inconsistencies among studies arise from philosophical discrepancies on the definitions. In this regard, we must recall that Rene Descartes laid down part of the philosophical foundation of contemporary science. He upheld the notion that the mind and the body may be treated as independent traits and thus proposed a machine metaphor for exploring the body's workings. Under his dichotomic view, comprehending emotions was an unnecessary element to understanding the body mechanisms of function or dysfunction. As a result, diseases are now perceived as the expression of the inadequate functioning of the body-machine, detached from the emotional world of the individual. Therapeutics, therefore, aims at correcting the body without considering the mindset and the emotional sphere. This is the philosophical background from which the first definition of stress emerged.

The basic premises of the physiological model of stress were truly articulated by Selye in 1936. From his perspective, stress is an organism's physiological response characterized by a general adaptation syndrome that results from the orchestrated activation of the nervous, endocrine, and immunological systems. As a consequence, under this conception, stress can only be diagnosed based upon shifts of several physiological parameters (e.g., blood pressure, body temperature, and cortisol plasma levels) [51]. Since psychosocial factors are now known to trigger or buffer stress responses, diagnosing it based only on physiological grounds is limited and inaccurate. Furthermore, the general adaptation syndrome model predicts the existence of two types of stress responses. In the acute stress response, the organism rapidly becomes adapted to the stressor(s), a circumstance that allows its return to the homeostatic state. In contrast, during the chronic stress response, the organism cannot develop adaptive response, so it does not return to the homeostatic state, leading in the long run to a deterioration of its health.

Twenty years later, Holmes proposed a second model in which stress was the byproduct of social circumstances that were inherently stressing. Under Holmes' view, the subject might encounter stressors during key moments of his life, thus conditioning his life's health, quality, and expectancy. Under this premise, Holmes proposed a list of stressors that he assumed were able to induce stress responses in most, if not all, individuals exposed to them. Although this concept was readily embraced by psychologists and in fact has inspired the construction of all psychological instruments currently used to estimate stress magnitude, the fact that (1) subjects may judge social circumstances as stressing or not depending upon their life experiences, (2) social interactions may buffer stress, (3) social behavior may change in response to stress, and (4) the psychological assessment of stress is not complemented by monitoring stress-related physiological variables makes stress diagnosis under Holmes' reasoning unsound.

Finally, in 1984, Lazarus and Folkman proposed a third conceptual model of stress. They postulated that for the physiological stress responses to proceed, the individual must first perceive circumstances as stressing. This assessment is made upon each subject's appraisal of his own abilities to cope with challenging circumstances. Although both premises have been welcomed by psychologists since they can be evaluated through psychological tests, the fact that (1) psychological assessments of stress are not complemented with physiological measurements, (2) stress coping mechanisms vary greatly among individuals, and (3) stress resilience also differs individually makes stress diagnosis based upon the Lazarus-Folkman premises imprecise.

**Figure 1:**
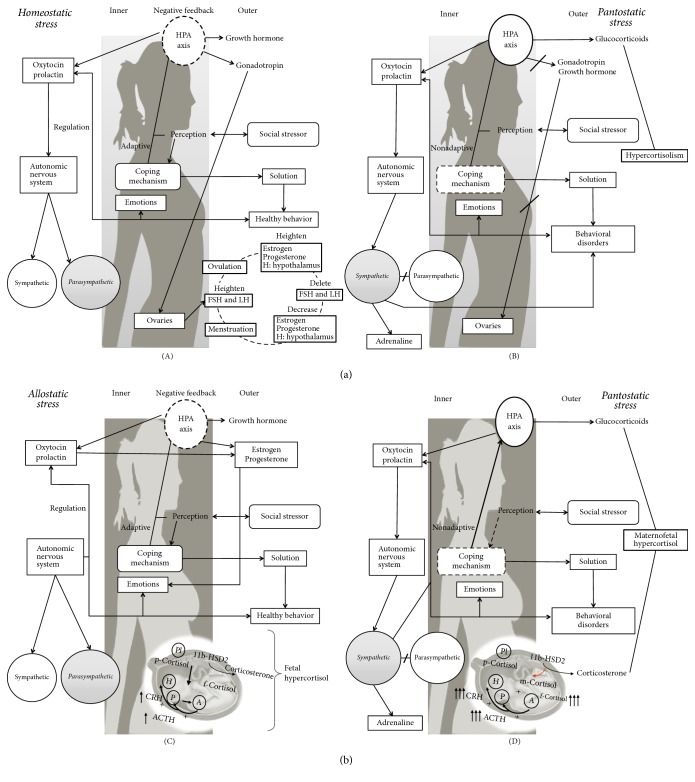
*Biopsychosocial stress in nonpregnant and pregnant women.* H: hypothalamus; P: pituitary; A: adrenal; PL: placenta; m, p, or f cortisol: maternal, placental, or fetal cortisol; p CRH: placental CRH; +: positive feedback loop; −: negative feedback loop.

As we can deduct from the preceding paragraphs, each of the stress models outlined failed to appreciate the contribution of different aspects of an individual's life to stress. It is precisely the absence of this integrated view of stress which makes its diagnosis in pregnant women problematic since their physiopsychosocial world differs significantly from the statistical norm set by nonpregnant, age matched women. In this regard, McEwen and Seeman (1999) (cited in [52]) introduced the term allostasis to refer to short term actions taken by the organism to adapt to the stressful situation. Even if allostasis works to restore the physiological balance, similarly to homeostasis, the difference between these two functional states is that homeostasis “seeks” to restore balance to the expected levels (i.e., temperature, pH, and hormone concentrations), while allostasis restores the physiological parameters to new set points, new parameters that allow the adaptation to the stressful event without compromising the organism's health. However, during stress allostatic responses, if the physiological adjustments are ineffective or inadequate and/or if the stressor(s) actions are long-lasting, the adaptation process is not achieved and the organism will then be facing an allostatic load [52]. Moreover, Campillo (2014) [53] introduces the term pantostasis to refer to the generalized physiological involvement that follows in response to situations that represent a global threat for the organism. Under pantostatic responses, the organism is unable to restore the homeostatic or allostatic balance thus leading to diseased states. In this context, it is clear that pregnancy may either impose homeostatic/allostatic (acute) or pantostatic (chronic) stress responses to women (Figure 1). In Figure 1, schemes illustrate the presumed interactions that are established among the biological (inner and outer), psychological, and social factors that influence stress perception in nonpregnant ((A) and (B)) and pregnant women ((C) and (D)): (A) the social and biogenic stressors perceived as challenging trigger a physiological acute stress response, which is modulated by positive coping mechanisms. This leads the HPA axis to have negative feedback in the glucocorticoids release, while growth hormones and gonadotropins are released. The gonadotropins hormones stimulate the sexual organs to start the women's reproductive cycle. At the same time the pituitary gland (part of the HPA axis) releases oxytocin and prolactin that influence the autonomic nervous system. These hormones modulate mood, anxiety, and depression and also promote the activation of the parasympathetic system thus facilitating relaxed mental states. All these responses are reflected in a healthy behavior that strengthens the promotion of a homeostatic stress. (B) In the opposite case, when the stress is perceived as potentially damaging and the coping mechanisms are inadequate or insufficient, the physiological stress response triggered surpasses the negative feedback control of the HPA axis. Therefore, the glucocorticoids released are unlimited, generating a hypercortisolism that inhibits the release of growth hormones and gonadotropins and in consequence the inhibition of the sexual cycle. Also in this pantostatic stress response, the oxytocin and prolactin release is suppressed, and there is a higher activation of the sympathetic system, which releases adrenalin. This situation is reflected in behavioral disorders that strengthen the pantostatic stress response. (C) In the pregnant woman, in whom there is already an ongoing allostatic stress response, the social and biogenic stressors perceived as challenging trigger a physiological acute stress response, which is modulated by positive coping mechanisms. The HPA axis retains a negative feedback, but there is a hypercortisolism because the placenta and the fetus release glucocorticoids. Under this circumstance, the placental 11*β*-hydroxysteroid dehydrogenase-2 (11*β*-HSD2) converts maternal cortisol into inactive corticosterone, thus protecting the fetus from acquiring hypercortisolism; the maternal cortisol can cross the placental barrier. This is accompanied by an increased release of oxytocin and prolactin that promotes the secretion of estrogen and progesterone, necessary to induce embryo/fetal immunotolerance. Oxytocin and prolactin promote positive emotions and later labor activity and the activation of the parasympathetic system. These responses are reflected in healthy behavior that modulates the allostatic stress. (D) In contrast, in pregnant women ongoing pantostatic stress, the social and biogenic stressors are perceived by them as damaging and their coping mechanisms are thus rendered insufficient to modulate the stress response. This circumstance deregulates the negative feedback on the HPA axis, thus leading to abnormal increase of cortisol levels that overpasses the ability of the placental 11*β*-HSD2 to buffer maternal cortisol, making it possible for the maternal cortisol to “overflow” the fetal circulation; excessive maternal cortisol can also downregulate placental 11*β*-HSD2 expression (discontinuous line), thus further decreasing the fetal protective effects of this enzyme. In addition, in the maternal and fetal brains, the cortisol-dependent negative feedback loop of the HPA axis tends to decay in strength (dashed lines) since glucocorticoid receptor expression diminishes in brain areas (e.g., hippocampus) that are critical to the attenuation of stress responses. As a result, the mother and the fetus fall into a relative state of hypercortisolism and HPA axis “sensitization.” This is even more critical because normal pregnancy is characterized by high cortisol levels due to the production of this hormone by the fetus and the placenta. Under these conditions, the abnormal hypercortisolism impairs the release of progesterone, estrogen, oxytocin, and prolactin thus facilitating the expression of negative moods. Also, the pantostatic stress in pregnant women enhances the activation of the sympathetic system leading to behavioral disorders that positively impact the pantostatic stress response.

## 3. The Pregnant Woman: Living under an Allostatic Stress Response

Pregnancy is a natural process that features physical, physiological, and psychological peculiarities embedded into an exceptional social context (Figure 1). The interrelation of these factors generally leads to allostatic stress and hence to a switching of the set points of a variety of physiological parameters. This is better illustrated by looking at the concentration of the key hormones of the HPA axis in the pregnant woman. Indeed, gestation features a progressive rise of cortisol, ACTH, and CRH serum levels from beginning to end [54, 55]. These increments result from the stimulation of placental release of CRH, which in turn promotes maternal and fetal cortisol production following the release of ACTH from the pituitary of both the mother and the fetus (Figure 1) [55, 56]. This physiological adjustment could easily be interpreted as stress if only diagnosed measuring the concentration of cortisol. However, whether this endocrine framework is pantostatic stress is debatable (see also [57]). During pregnancy, chronic hypercortisolism occurs as a biological demand of the fetus, since glucocorticoid elevations promote fetal programming and maturation [10, 58–61] and embryo/fetal immune tolerance [32, 61–67]. That is, increases of the HPA hormones serum concentrations may indicate that the mother's organism is adjusting her metabolic condition to an updated threshold for the time of pregnancy; at best it reflects an allostatic stress response. Thus, since the set point of the HPA axis is raised during pregnancy, measurements of HPA hormones as stress indicators may be misleading, since normal concentrations during pregnancy have not been established. In fact, it has been proved that the hormonal response of the HPA axis and the response of the sympathetic nervous system to emotional and physical stressors are severely attenuated during pregnancy [68]. These complex adaptations of the maternal brain are likely to be a consequence of an increased activity of the brain systems with inhibitory effects on the HPA axis (such as the oxytocin and prolactin systems) and of a reduced activity of the excitatory pathways (noradrenaline, corticotrophin-releasing factor, and opioids [68]).

A similar allostatic adjustment of set points occurs with sex steroid concentrations. During gestation, there is also a significant, progressive increase in progesterone and estradiol that keeps, during the first weeks, the embryo implanted by avoiding endometrial shedding.

Other allostatic adjustments occur in other spheres of the life of pregnant women. For instance, increased appetite is common among them. It is presumed that this change permits the adequate allocation of resources for building up the body of both the mother and her developing child during pregnancy [69]. Also, pregnant women experience social and emotional ambiguity and imminence states, with mixed feelings of happiness, insecurity, and fear that lead to a big desire for support by their partner and/or family. Thus, the biopsychosocial world of the pregnant woman indeed sets the stage to develop allostatic responses to circumstances perceived as stressing.

## 4. An Integrated Model of Stress: The Proposal

**Figure 2:**
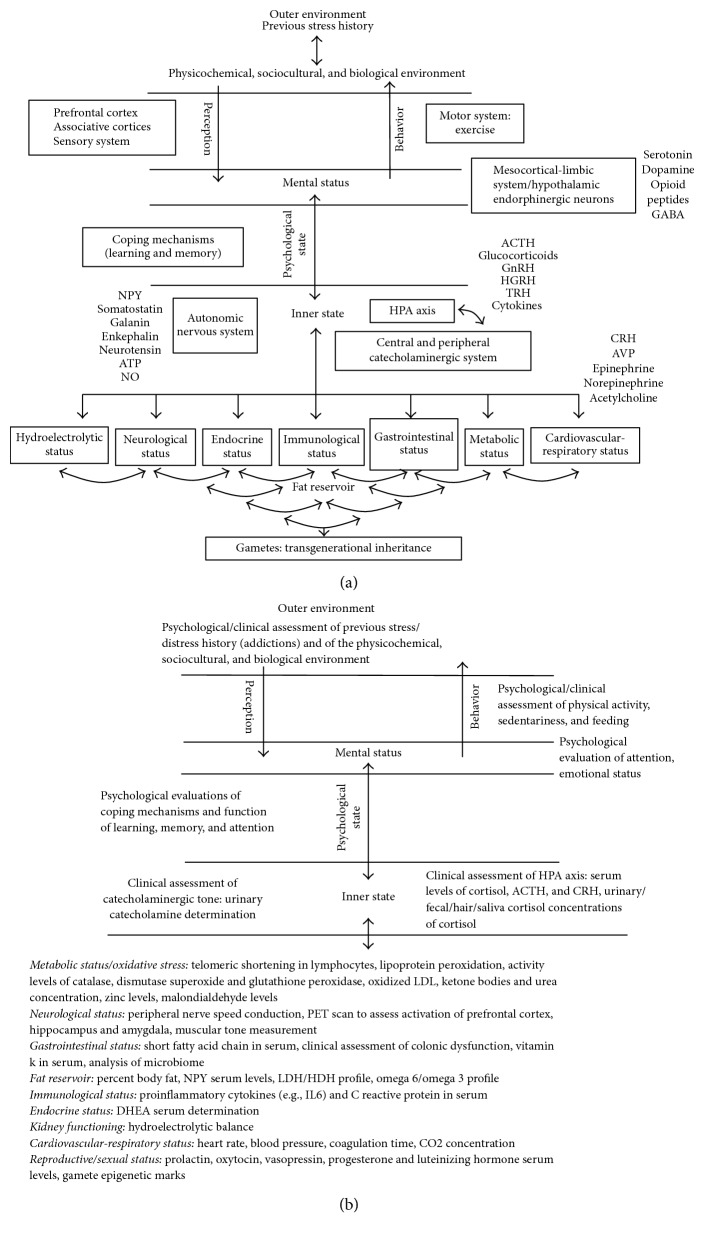
*Models to assess stress. (a) An integrated model of stress.* This layered model depicts the levels of organization, organic systems, and factors that might interact with one another within a multidimensional framework to sustain stress responses. In this context, if a handful of the elements depicted are acutely challenged (e.g., thirst stress response), the organism will only require a homeostatic response while keeping the set points of the parameters involved in such a response relatively unchanging. In contrast, if most body systems are acutely, subacutely, or chronically challenged (e.g., normal pregnancy), the organism would be forced to develop an allostatic response. This circumstance would provoke shifts in the set points of the parameters involved in such a response. Finally, if the entire body is acutely, subacutely, or chronically challenged (e.g., true gestational stress), the organism would be forced to mount a pantostatic stress response falling into a state of deregulation and disease.* (b) Protocol to evaluate stress in the pregnant woman.* This layered model of diagnosis, backed up by the model of stress, described above, aims to protocolize the psychological, clinical, and biochemical tests that must be used to effectively diagnose stress during pregnancy, assuming this as an allostatic challenge to the mother embedded into the multidimensional framework that sustains stress.

From our perspective, the concept of stress must be embedded in a “layered” model in which the elements/factors composing it interact, directly or indirectly, within and across levels of organization (Figure 2). In our model, the outer environment provides physicochemical, sociocultural, and biological information that is perceived by the subject through the combined functioning of the sensory systems, associative cortices, and prefrontal cortex. Environmentally derived informational clues may shift the individual's mental status if these are perceived as stressing after being “introjected” in the psychological state through the mesocortical-limbic system (i.e., ventral tegmental area, cerebral cortex, amygdala, and hippocampus) and by the hypothalamic endorphinergic neurons. Introjections will be considered as stressing only after estimating the ability to cope with them based upon merging the incoming information with the information retrieved from our memories/experiences through mental processes. If circumstances are judged as stressing, the stress-related psychological state is translated into a physiological stress response by the combined effects of diverse mediators released by the autonomic nervous system (i.e., adrenergic nerves), the central (i.e.,* Locus coeruleus*) and peripheral catecholaminergic system (i.e., chromaffin cells of the adrenal medulla), and the hypothalamus- (i.e., CRHergic neurons) pituitary- (i.e., corticotrophs) adrenal cortex (HPA) axis [70]. The stress response might be homeostatic if developed during short time windows and if it involves only a subset of organic systems. In the case where the stress response remains for longer time windows, the organism might reset the set points for a number of physiological variables, a circumstance that would lead to allostatic stress. If stress acquires a truly chronic status, resetting set points of some variables will not suffice to confront the challenge. The organism would then be forced to mount a pantostatic stress response, a condition that involves the entire body [71, 72]. In every case, the internal milieu generated by the different types of stress responses would be fed back to the psychological and mental status leading to the expression of adaptive or nonadaptive behaviors through a specific motor output that in turn would influence the environment. Overall, the multidirectional interaction among all these layers would lead, when working under the “acute” mode, to increased arousal and attention, suppression of pain sensation, decreased appetite, and increased thermogenesis. When the stress system works under the “subacute” and “chronic” modes, it would induce anxiety, depression, decreased thyroid function, impaired growth, and suppressed sexual and reproductive functions via decrements of the catecholaminergic tone. It would also promote visceral adiposity, insulin resistance, dyslipidemia, hypertension, and osteoporosis. Finally, the CRH produced by central neurons would inhibit systemic inflammatory reactions via glucocorticoids and catecholamines, while the CRH release by peripheral nerves would stimulate local inflammation [70].

Ultimately, the proposed model incorporates, on one hand, the influence of the previous stress history as a pervasive factor that conditions the way the subject will ponder, interpret, and cope with potentially stressing circumstances. On the other, it considers the possibility of inheriting “stress-conditioned” phenotypes to the offspring via epigenetic mechanisms working on the gametes genome.

## 5. Estimating Stress during Pregnancy

Acute and chronic stress are multidimensional concepts that our group operationally defines as the physiological state that results from an unfavorable perception of one's capacities/abilities to cope with challenging environmental demands. Since stress perception is a highly subjective individual experience and involves the assessment of biological, psychological, and social aspects of living [45, 73], assessing it becomes extremely challenging.

In spite of these difficulties, psychologists have designed a number of instruments intended to estimate the magnitude of the perceived stress [45, 74]. Table 1 shows the psychological instruments that are most frequently used to assess stress during pregnancy and its associations with its outcome and with cortisol level measurements (Table 1). Notice that there are discrepancies among the studies. Some of them reported positive correlations among psychological estimations of stress, pregnancy outcome, and cortisol serum levels while others did not. This might be attributed to the inadequacy of the psychometric instruments used to estimate stress. For example, of fifty-seven studies only nineteen used instruments to assess stress. Thirty-eight studies employed anxiety and depression tests like the* State-Trait Anxiety, *the* Pregnancy Specific Anxiety Scale*, and the* Center for Epidemiological Studies Depression Scale* to estimate stress. Also, from Table 1, the most widely used instrument is the* Perceived Stress Scale*, an instrument designed to estimate stress at any time point of life but with no consideration for the emotional and social status of pregnant women. Actually, from the studies listed, only one used a specific instrument to measure stress during pregnancy. Even though both emotional states commonly result from the interpretation of daily life circumstances as stressing [75], anxiety and depression are not emotional states exclusively expressed under stressing conditions. Under these circumstances, cortisol might be precise biomarker of stress since it normally increases during pregnancy. Additionally, although several studies show associations between stress and pregnancy outcomes (i.e., effects in infant development, low birth weight, preterm delivery, preeclampsia, abortion, and emotional disorders), these same associations were observed in pregnant women with anxiety and depression. It is interesting to note that only in the case of preterm delivery was the correlation with stress higher than that associated with anxiety or depression. Once again, in spite of these limitations, instruments that evaluate the state/trait parameters of anxiety and depression have been used to quantify “maternal stress” in humans. Thus, the above-mentioned instruments might be insufficient to estimate stress in pregnant women. Moreover, pregnant women can also express anger, apathy, and avoidance after interpreting diverse circumstances as stressing; instruments must also explore these emotional states. Another problem shared by the psychological instruments designed to estimate stress levels in pregnant women is that most of them (if not all) were not primarily designed to explore pregnant women. In this regard, Alderdice et al. (2012) [19] propose that one of the main constraints of psychometric instruments is precisely that they are not adapted to the study population; that is, cultural aspects are not taken into account, and neither are the changes brought by the pregnancy stages. Another important issue is the theoretical basis in which the instruments were built. For example, most of the instruments to assess stress in pregnancy come from events that are commonly considered as stressful, in which stress is a consequence of the quantity of environmental changes that are perceived by the person [76]. This model neglects the person's skills to cope with and manage stress. The women's skills to cope with stress are very important since several studies have shown that not all pregnant women who report high levels of stress go on to develop complications [77, 78]. With regard to this issue Nierop et al. (2008) [78] found that some psychosocial resources such as self-efficacy and daily uplifts promote positive outcomes during pregnancy and* postpartum*. For example, when pregnant women have high levels of psychosocial resources, they perceive low stress and their cortisol and alpha-amylase levels are also lower [78]. In this regard, the questionnaires may explore issues that might be irrelevant for pregnant women leading to stress over/underrating [79].

Another source of unreliable data and misinterpretations on the effects of stress during pregnancy is the diversity of techniques used to estimate glucocorticoid concentration. Indeed, researchers use different methods to measure glucocorticoids from saliva, urine, feces, or hair [80, 81] assuming they all are good indicators of glucocorticoid plasma levels. Studies have shown, nonetheless, that such assumption might not be solidly grounded since there seem to be no direct correlations between these parameters. Table 1 summarizes the main research linking stress during pregnancy with physiological measures of stress: cortisol concentrations. More discrepancies were found in this regard; from the 21 papers reviewed, 50% found an association between gestational stress and levels of cortisol but only in the first trimester of gestation. For instance, de Weerth and colleagues (2003) [1] found that women with higher levels of cortisol during late pregnancy delivered their infants earlier than those with lower values. Even so, to associate high levels of cortisol with stress and stress responses during pregnancy has not been an easy task; Salacz et al. (2012) [26] did not find cortisol as a significant predictor on psychometric scores even when they found high levels of perceived stress and fear of delivery, perhaps because they analyze stress in the last trimester of gestation, while other studies did not find these associations; for example, Romo-González et al. (2012) [34] did not find correlations between plasma cortisol concentrations and low birth weight in children's from women with moderate anxiety. Similarly, another study conducted by Kramer et al. (2009) [7] found no associations between cortisol concentrations in hair and premature delivery in women under stress during pregnancy. These contradictions could be the consequence of differences in the gestational period in which the physiological measures were performed and/or the time at which the sample was carried out (it is known that there are variations in glucocorticoid concentrations throughout the day) [82].

Given that pregnancy is a process that imposes an allostatic stress response in which the entire organism needs to generate adjustments in order to allocate resources for her and her child, the diagnosis of stress becomes a challenge. Clearly, norm values of various parameters considered as relatively precise indictors of stress are inappropriate since they are likely to represent homeostatic responses. Allostatic values of the parameters monitored should be taken as references to diagnose stress in pregnant women. The risk for a pregnant woman is to function under stress, since their bodies are already functioning under allostatic stress. The real risk comes from the possibility of them developing pantostatic stress syndromes such as eclampsia. In this context, a handful of measurements might not be sufficient to monitor the risk of a pregnancy transitioning from allostatic to pantostatic stress. We believe an increased number of tests must be used in order to have a better picture of the levels of stress during pregnancy. This argument is valid for both psychological and biochemical determinations.

In Figure 2, we propose a protocol to be used to evaluate stress in the pregnant woman. We assumed that reference values were taken not from nonpregnant women even if they had ongoing homeostatic stress. Reference values must be estimated based on measurements obtained from pregnant women coursing normal, allostatic pregnancies.

### 5.1. Clinical and Psychological Evaluations

Clinical and psychological evaluations must provide qualitative and quantitative information on previous stress history; that is, the assessment of stress during pregnancy should not ignore the fact that pregnant women are engaged in a social environment that constantly bombards her with stressors. According to Holmes' proposal, the more the stressors that are perceived, the higher the stress response.

**Figure 3:**
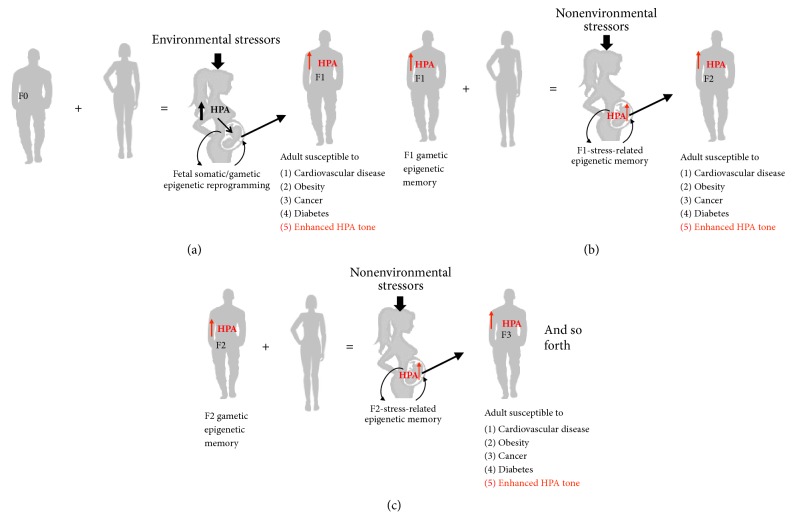
*Schemes that illustrate the hypothetical process underlying the transgenerational epigenetic inheritance of stress susceptibility and stress-related diseases via the paternal lineage. *(a) When an “unstressed healthy couple” decides to become pregnant, the “biological future” of their child (and indeed of their lineage) might be deeply influenced by the circumstances that surround their pregnancy. In the case where the pregnant mother (F0) experiences stress, the ontogenetic trajectory of the fetus may deviate his/her phenotype to an alternative form that renders the child, and later the adult, susceptible to develop degenerative diseases (see Figure 2) and altered HPA axis physiology (see Figure 1), respectively. The alternative phenotype, however, features increased susceptibility to develop not only stress-related diseases and “stress hypersensitivity” but also spermatogonia that kept a record of the prenatal stress episode in their genome through epigenetic memories. (b) When the spermatozoids derived from the stress-modified precursors of the F1 male fertilize the oocyte of a “naïve” woman, even if the pregnancy proceeds under “no-stress conditions,” the conceived child (F2) inherits the “stressful phenotype” due to the permanence of the F-1 stress-related, gametic epigenetic memory. (c) A similar process takes place in subsequent generations. There is information supporting that this may happens for at least five generations. Epigenetic gametic inheritance can also applied to the maternal lineage, and it is possible that if both members of the couple were exposed to stress during fetal life, their gametes could “double the dosage” of the stress-related epigenetic memories passed on to their descendants making them much more stress prone and stress susceptible throughout their lives.

Moreover, it is also important to consider the stress that the women's parents experienced in their life course and, if possible, request information about the stress that her mother experienced during pregnancy. This is very important since, as will be shown later, stress is inherited; this will give us information on the person's susceptibility to stress (Figure 3).

One of the limitations that have been observed in psychometric instruments is that the aspects that could be perceived as stressful during pregnancy are rarely taken into account and that these stressors may be varied according to location, culture, and age. This is a key point, since on one hand the more the aspects are perceived as stressors, the more stressful the stress response is (according to the Holmes theory) and on the other hand the fact that pregnancy is perceived or not as stressful will trigger or modulate the stress response and coping mechanism (according to the Lazarus and Folkman proposal). The latter is very important since the coping mechanisms link the stressors from the environment with the physiological response of the organism to stress (Figure 1).

Another aspect to consider is the type of personality. Several studies show that people who are characterized as being competitive, overachieving, pressured by time, impatient, and hostile respond more readily to neutral or ambiguous situations as stressful [83]. The type of personality also determines the coping mechanism that the individual uses in response to stress. For example, it has been observed that people under stress tend to consume sweet and fat food with high caloric content (comfort food), due to their effects in the sensation of pleasure and relief from the stress; they also perform less physical activity. Thus it is also important to consider eating and physical activity habits [84, 85] in the elaboration of a psychometric instrument that is intended to assess stress during pregnancy.

In summary, a more accurate assessment of stress during pregnancy must consider the interaction of all these points, which in turn allows an estimation of the magnitude of stress and an understanding of how all the clinical and psychological aspects affect, directly and indirectly, the modulation of the physiological response to stress.

### 5.2. Biochemical Determinations

Since allostatic stress associated with pregnancy involves adjustments in most if not all of the organic systems, the precise diagnosis of stress through biochemical determinations must evaluate stress manifestations in most of those systems. Hence, telomeric shortening in lymphocytes, lipoprotein peroxidation, activity levels of catalase, dismutase superoxide and glutathione peroxidase, and oxidized LDL could provide information on the oxidative stress, commonly increased when passing from allostasis to pantostasis. Glucose, ketone body, and urea concentrations would indicate carbohydrate, lipid, and protein consumption and degradation. Peripheral nerve speed conduction, PET scans to assess activation of prefrontal cortex, hippocampus and amygdala, and muscular tone measurements could provide information on neuromotor function. The determination of short-chain fatty acid in serum, vitamin K, and a clinical assessment of colonic dysfunction would allow gastrointestinal evaluation. The percentage of body fat, NPY serum levels, LDH/HDH, and omega 6/omega 3 indices would help estimate the atherogenic and obesity potentials. Proinflammatory cytokine profiling and protein C immunoreactivity could establish whether an inflammatory process is ongoing. DHEA is a steroidal hormone that protects against stress. Its determination would allow us to estimate the risk of stress damage. Other parameters such as heart rate, coagulation time, CO2 concentration, the availability of prolactin, oxytocin, opioids, progesterone, estradiol, luteinizing hormone, and vasopressin, and the hydroelectrolytic balance would provide indicators of the cardiovascular, respiratory, reproductive/sexual, and renal function. Finally, epigenetic mark profiling could advise on the possibility of gametes affection.

We believe that combining clinical, psychological, and biochemical studies aimed at evaluating as many parameters as possible would increase our chances of having a precise chronic stress diagnosis. This approach would surely solve the controversy on the effects of gestational stress on the mother/child health in both the short and long term.

While it appears that the parameters to be considered in the diagnosis of chronic stress are excessive, we think this approach is ideal since it allows for an evaluation of various aspects of the functions of the body under the assumption that stress responses may involve most physiological systems.

## 6. Previous Stress History and Transgenerational Inheritance of “Stress-Conditioned” Phenotypes as a Key Factor to Take into Account during Stress Assessment

A final word on important aspects that are rarely considered when assessing gestational stress: When we talk about the impact that a life record has on any process, we commonly think that past and current circumstances might have conditioned actual and future outcomes. Most research evaluating the effects of stress during pregnancy does it when stressful conditions are already affecting the pregnant mother (Figure 3) [60, 62, 86–94]. What has not been evaluated thoroughly is how the stress experienced by the future mother during her life from conception to the moment of becoming pregnant may condition her well-being during and after pregnancy and, more importantly, the prenatal and postnatal development and health of her future child, grandchildren, and forthcoming generations. One may have hints that such a conditioning occurs based upon observations made in both animal models and humans.

One of the most consistently adverse effects of chronic stress is disruption of reproductive physiology and behavior [95]. As Winglfield and Salpolsky (2003) [92] wisely said “Whether one is a clinician trying to understand a patient's loss of libido, a wildlife biologist grappling with how habitat degradation translates into decreased fertility of wild populations, or a conservationist faced with an endangered species refusing to mate in a zoo enclosure, stress must be considered in the equation.” For example, anybody familiar with rodents' reproduction may have experienced the difficulties of getting female rats and mice pregnant when they are subjected to stressful conditions (e.g., loud ambient noise, sleep deprivation, and social stress), prior to copulation [95, 96]. A similar story has unfolded for humans in recent years (e.g., [97]). Preconception stress is also known to shift sex ratio both in experimental animals and in humans (e.g., [98]).

The impact of the life record of stress on future mothers' phenotypes/genotypes is not restricted to fertility issues and to differing sex ratio of the offspring. Now we know that if the mother (grandma) experienced stress during pregnancy, her daughter (the mom; F1 generation) will epigenetically inherit the “grandma stress” even if she does not experience stress of a similar sort [99–102]. Work amassed during the last couple of years supports that gametes-mediated transgenerational epigenetic stress inheritance is a process that can go on for at least four generations and influence various phenotypic traits in several peripheral tissues and the brain, including the HPA axis form and function [102], in both male and female individuals along the family's linage (Figure 3) [103, 104]. This transgenerational epigenetic stress includes all kinds of risks from cardiovascular or metabolic diseases to psychiatric disorders [99–102]. Moreover, transgenerational epigenetic stress inheritance is not a phenomenon circumscribed to the feminine gender. Recent studies support the fact that spermatozoa could also seed stress epigenetic memoirs upon the genome of various individuals across generations [50, 86–94]. It is interesting that some reports found that there are sex differences in prenatal epigenetic programing of stress pathways. For example, Mueller and Bale (2008) [105] found that stress early in pregnancy increased physiological and behavioral stress responses specifically in male offspring as adults. Also epidemiological studies linking fetal antecedents with long term disease risk have established that gender is an important determinant in disease severity and onset, as it has been proved by van Os and Selten (1998) [90] in the offspring of pregnant mothers exposed to the stress of the 1940 invasion of the Netherlands, in which male but not female had an increased risk for schizophrenia as adults.

Additionally, while most maternal exposures are examined during pregnancy and thus have the ability to affect more directly the somatic development of her offspring, paternal studies have proved that it is able to pass on information via germ cells. So, if the male offspring are programmed differently as a result of their life experience (i.e., stress, malnutrition, among others), then that information is present in his sperm [100].

Hence, future studies must take into account a recollection of data in the stress life history of the mother and, ideally, of the couple.

## 7. Conclusions and Future Directions

For many years, biomedical scientists have affirmed that stress during pregnancy leads to various perinatal health complications and predisposes both the mother and her children to develop diseased states later in postnatal life. Evidence contradicting this view has shed doubts on this seemingly straightforward conclusion. Although these authors support the notion that gestational stress deleteriously affects the health of the mother and her child in the short, intermediate, and long term, we also recognized the lack of consistency of the reported results. We believe that the origin of all these contradictions lies in the way that stress has been conceived throughout history; that is, the approach has generally ignored the interaction of the biological, psychological, and social human counterparts. This lack of integration had led to the absence of information of both parents' life stress record, to name a few. Another important point to consider is that the pregnancy itself promotes a series of changes which result in allostatic stress. The failure to consider pregnancy as an allostatic stress condition has led to misunderstanding of the meaning of HPA axis activation during pregnancy and to inaccurate estimates of stress with psychological instruments designed to assess other psychic conditions (primarily anxiety and depression) or designed to measure the stress perceived in open populations who have not experienced stress during pregnancy.

To overcome these shortcomings, we believe that future studies must consider the dynamic nature of the HPA axis in pregnant women, understanding its true nature with its positive faces such as fetal programming and maturation [10, 58–61] and embryo/fetal immune tolerance [1, 32, 61–65], that is, the recognition of the allostatic stress condition that promotes pregnancy and a new stress model that takes into account the interaction of what is perceived as stressful by the pregnant woman, the coping mechanisms which are launched, and the stress-related physiological states (Figure 2). This complex stress-related physiological state could not be assessed with cortisol as the unique biomarker. Other hormonal/nonhormonal stress-related markers (i.e., insulin, catecholamines serum concentrations, and the activity of saliva enzymes, among others) considered to be surrogate markers of sympathetic activation in response to stress in samples obtained from a variety of tissues (e.g., stools, hair, and saliva) should be considered to ensure that current noninvasive methods to estimate steroids are reliable and unbiased predictors of stress-related hormones in blood in both the short and long term and perhaps, more importantly, to design adequate psychological tests that could specifically estimate stress in pregnant women and/or in women that plan to become pregnant based on their specific challenges and expectations and to thoroughly detail the couple's stress family record.

Finally, it is important to highlight that in order to have a good diagnosis of the magnitude of stress during pregnancy, previous history of stress and epigenetic markers, personality type, eating, and physical activity habits are also to be taken into account.

## Figures and Tables

**Table 1:** *Stress during pregnancy, cortisol levels, and its association with pregnancy outcome*. Adverse outcome of stress for the pregnancy was reviewed in the latest bibliography in order to know the type of association that stress causes on pregnancy and the type of mood disorder (anxiety and depression) which were the tools applied to assess stress during pregnancy. In italics the adverse outcomes related to stress and depression are shown, while in bold those adverse outcomes among anxiety or the combination of the mood disorders are shown. On the psychometric test column the double asterisks (*∗∗*) show those studies in which an inventory of stress was used. From [12–17] those researches used on animal models to study the prenatal stress long term effects on the offspring are obtained. Besides this table shows the studies assessing physiological stress with cortisol measurements; different tools were found along the studies and different trimester of gestation.

Reference	Stress and adverse pregnancy outcome		Association with cortisol		Type of adverse outcome	Mood variables	Tools assessing stress *Psychometric test/biomarkers*	Gestational age	Studies with human or animal
	Yes	No	Yes	No					
[15] *Huizink et al. (2002)*	*X*				*Infant temperament at 3 and 8 months of age*	*Stress*	^*∗∗*^ *Stress: perceived stress and pregnancy anxiety* *Infant: direct observation and by parent report*		**Human**

[23] *DiPietro et al. (2002)*	*X*				*Fetal motor activity*	*Stress*	^*∗∗*^ *Affect Intensity Measure, Daily Stress Inventory, Pregnancy Experience Scale* *Fetal heart rate, fetal movement*		*Humans*

[1]* De Weerth et al. (2003)*	*X*				*Premature birth*	*Stress*			**Humans**

[2] *Sandman et al. (2006)*	*X*				*Preterm birth (19 weeks of gestation)*	*Hormonal stress*	*CRH and plasma cortisol*		**Human**

[3] *Nepomnaschy et al. (2006)*	*X*		*X*		*Spontaneous miscarriages*	*Hormonal stress *	*Cortisol in urine*		**Human**

[4]* Grandi et al. (2008)*	*X*				*Preterm birth*	*Stress*	*Focus group and psychosociocultural interview*		**Human**

[5]* Lobel et al., (2008)*	*X*				*Preterm birth*	*Pregnancy specific stress*	^*∗∗*^ *Prenatal Distress Questionnaire, Prenatal Life Events Scale, State Anxiety Subscale of the State-Trait Personality Inventory, Prenatal Health Behaviors Scale*		*Humans*

[7]* Kramer et al. (2009)*		*X*		*X*	*Preterm birth*	*Stress*	^*∗∗*^ *Perceived Stress Scale, Pregnancy-Related Anxiety by Dunkel-Schetter, Rosenberg Self-Esteem Scale, Life Orientation Test, Center* *for Epidemiologic Studies Depression* *CRH, placental histopathology and maternal hair cortisol*		**Human**

[26]* Salacz et al. (2012)*	*X*			*X*	*Anxiety and depression*	*Feelings of distress*	^*∗∗*^ *Beck Depression Inventory, State-Trait Anxiety Inventory, Perceived Stress Scale, a Likert-like scale for fear of delivery, a structured interview to assess health and socioeconomic status Plasma cortisol*		**Human**

[27]* Buss et al. (2012)*	*X*		*X*		*Emotional problems in children*	*Hormonal stress *	*Child Behavior Checklist* *Cortisol in saliva*	*15 weeks of gestation*	**Human**

[14]* Butler et al. (2002)*	*X*				*Fetal programming, neurological disorders, psychiatric disorders, and metabolic and cardiovascular problems*	*Stress*	*Glucocorticoid*		**Animals**

[28] *Van den Hove et al. (2005)*		*X*			*Anxiety and depressive-like behavior in the offspring*	*Stress*	*The open field test, the home cage emergence test, and the Forced Swim Test (offspring)* *Plasma corticosterone (pregnancy)*		**Animals**

[29] *Kraszpulski et al. (2006)*	*X*				*Differences in amygdalar nuclei, suggesting that this may predispose to fear related behaviors such anxiety*	*Stress*			**Animals**

[30] *Yang et al. (2006)*	*X*				*Altering synaptic plasticity and enhancing the effects of acute stress on synaptic plasticity in the hippocampus, which may be the mechanism for the impaired spatial learning and memory in young rat offspring*	*Stress*	*Morris water maze*		**Animals**

[31] *Pawluski et al. (2011)*	*X*				*Increased anxiety-like behavior, decreased* *depressive-like behavior, and lower corticosterone levels*	*Stress*	*Elevated Zero Maze (anxiety), Forced Swim Test (depression)*		**Animals**

[18] *Howerton and Bale (2012)*	*X*				*The male offspring displayed anhedonia and increased sensitivity to selective serotonin reuptake inhibitor* *treatment. Long term alterations in central corticotropin-releasing factor (CRF) and glucocorticoid receptor (GR) expression, as well as* *increased hypothalamic*-*pituitary-adrenal (HPA) axis responsivity, were present in these mice and likely contributed to an elevated* *stress sensitivity*	*Stress*	*Comparisons between placental PCR array and epigenetic analyses*		**Animal**

[32] *Brunton and Russell (2008)*	*X*				*Low weigh and hyperinsulinaemic at 6 months of age following oral glucose load in the female and high blood glucose in adult males*	*Stress*			**Animals**

[33] **Orr et al. (2007)**	**X**				**Preterm birth**	**Anxiety**	**Prenatal Social Environment Inventory**		**Human**

[7] **Kramer et al. (2009)**	**X**				**Preterm birth**	**Anxiety**	^**∗∗**^ **Perceived Stress Scale, Pregnancy-Related Anxiety by Dunkel-Schetter, Rosenberg Self-Esteem Scale, Life Orientation Test, Center for Epidemiologic Studies Depression** **CRH, placental histopathology and maternal hair cortisol**		**Human**

[34] **Romo-González et al. (2012)**	**X**				**Pregnancy complications, hypertension, and preterm birth**	**Anxiety**	**State-Trait Anxiety Inventory** **Serum cortisol, estradiol, and progesterone**		**Human**

[34] **Romo-González et al. (2012)**		**X**			**Low birth weight**	**Anxiety**	**State-Trait Anxiety Inventory** **Serum cortisol, estradiol, and progesterone**		**Human**

[35] *Steer et al. (1992)*	*X*				*Preterm birth and low birth weight*	*Depression*	*Beck Depression Inventory*		**Human**

[9] *Field et al. (2004)*	*X*				*Preterm birth and low birthweight* *The newborn's biochemistry (except for epinephrine) was higher than the maternal biochemistry*	*Depression*	*Urine cortisol, norepinephrine, serotonin, epinephrine, and dopamine*		**Human**

[36] *Diego et al. (2009)*	*X*				*Preterm birth and low birth weight*	*Depression*	*Structured Clinical Interview for DSM-IV Axis I Disorders, The Center for Epidemiological Studies Depression Scale* *Urine cortisol*	*18 weeks of gestation*	**Human**

[37] *Hompes et al. (2012)*	*X*		*X*	*X*	*Low birth weight*	*Depression*	*Anxiety and depression* *Basal cortisol*	*Only first trimester but not third trimester*	**Human**

[38] **Wadhwa et al. (1993)**	**X**				**Birth weight and gestational age at birth**	**Stress and anxiety**	^**∗∗**^ **Perceived Stress Scale, Hopkins Symptom Checklist, an instrument** **assessing maternal fears and anxiety related to the** **health of the baby, toward the labor and delivery process,** **and confidence in the obstetrician and other** **health care providers,** **1- and 5-minute Apgar scores**		**Human**

[39] **Brouwers et al. (2001)**	**X**				**Child development (three weeks postpartum and after 2 years)**	**Stress and anxiety**	**State-Trait Anxiety Inventory** **Neonatal Behavioral Assessment Scale** **Bayle Scales of Infant Development**	**32 weeks of gestation**	**Human**

[40] **Rieger et al. (2004)**	**X**				**Child development (three weeks postpartum and after 2 years)**	**Stress and anxiety**	**Neonatal Behavioral Assessment Scale**		**Human**

[6] **Harville et al. (2009)**	**X**			**X**	**Preeclampsia**	**Stress and anxiety**	^**∗∗**^ **Perceived Stress, state-trait anxiety coping style, life events, social support, and pregnancy specific anxiety** **Plasma cortisol and CRH**		**Human**

[41] **Davis and Sandman (2010)**	**X**		**X**	**X**	**Elevated concentration of cortisol early in gestation was associated with a slower rate of** **development over the first postnatal year and lower scores on the mental development index of the** **BSID at 12 months** **Elevated levels of maternal cortisol late** **in gestation were associated with accelerated development over the first year and higher scores on the BSID** **Elevated levels of maternal pregnancy specific anxiety early in pregnancy were independently associated with lower scores on the BSID at 12 months**	**Stress and anxiety**	**Bayle Scales of Infant Development (BSID), Mental Development Index and Psychomotor Development Index** **Salivary cortisol**	**Only first trimester but not third trimester**	**Human**

[42] **Paarlberg et al. (1999)**	**X**				**Low birth weight**	**Stress and depression**	^**∗∗**^ **Daily stressors, psychological and mental well-being**		**Human**

[43] **Davis et al. (2004)**	**X**				**Infant negative behavioral reactivity**	**Anxiety and depression**	**State-Trait Anxiety Inventory and Center for Epidemiological Studies Depression Inventory** **Harvard Infant Behavioral Reactivity Protocol**	**32 weeks of gestation and 8 weeks after delivery**	**Human**

[44] **Pimentel (2007)**	**X**				**Urinary infection (anxiety), preterm birth (depression), and preeclampsia (family dysfunction)**	**Anxiety and depression**	**Test Goldberg (anxiety and depression) and APGAR scale (Appearance, Pulse, Grimace, Activity, Respiration)**		**Human**

[8] **Diego et al. (2006)**	**X**		**X**		**Low fetal weight**	**Stress, depression, and anxiety**	^**∗∗**^ **Daily hassles, Center for Epidemiologic Studies Depression, and State-Trait Anxiety Inventory Scales** **Urine cortisol and norepinephrine**	**Half of gestation**	**Human**

[12] **Vianna et al. (2011)**	**X**				**Preeclampsia**	**Stress, depression, and anxiety**	**Testing through in vitro proliferation assays** **Testing through in vivo approaches**		**Humans**

[45] **Ruiz et al. (2003)**				**X**	**Stress**	**Plasma cortisol**	^**∗∗**^ **Perceived Stress Scale**	**All gestational period**	**Human**

[46] **Sarkar et al. (2007)**			**X**		**Stress**	**Plasma cortisol and amniotic fluid**	**Not specified**	**17 to 18 weeks onwards**	**Human**

[47] **Buske- Kirschbaum et al. (2007)**			**X**		**Stress in preterm birth children**	**Salivary cortisol**	^**∗∗**^ **Psychological Stress Test**		**Human**

[48] **Entringer et al. (2010)**			**X**		**Stress**	**Blood pressure, heart rate, and salivary cortisol**	**Trier Social Stress Test (TSST), ** ^**∗∗**^ **Perceived Stress Scale, and Center for Epidemiological Studies Depression Scale**	**Only first trimester but not third trimester**	**Human**

[49] **Kaasen et al. (2012)**				**X**	**Stress**	**Salivary and serum cortisol**	**Impact of Event Scale, General Health Questionnaire, and Edinburgh Postnatal Depression Scale**	**12–32 weeks of gestation**	**Human**

[50] **Voegtline et al. (2013)**				**X**	**Stress and well-being**	**Salivary cortisol**	**Spielberger Trait Anxiety Scales, Center for Epidemiologic Survey Depression Scale, Pregnancy Experiences Scale, World Health Organization Well-being Index Five**	**Second half of gestation**	**Human**
